# Sex and race/ethnic disparities in the cross-sectional association between depressive symptoms and muscle mass: the Multi-ethnic Study of Atherosclerosis

**DOI:** 10.1186/s12888-015-0604-9

**Published:** 2015-09-18

**Authors:** Rosemay A. Remigio-Baker, Matthew A. Allison, Pamela J. Schreiner, Mercedes R. Carnethon, Jennifer A. Nettleton, Mahasin S. Mujahid, Moyses Szklo, Rosa M. Crum, Jeannie-Marie Leuotsakos, Manuel Franco, Nicole Jensky, Sherita Hill Golden

**Affiliations:** Department of Epidemiology, Johns Hopkins Bloomberg School of Public Health, 615 N. Wolfe Street, Baltimore, MD 21205 USA; Department of Family Medicine and Public Health, University of California San Diego, 9500 Gilman Drive, La Jolla, CA 92093 USA; Division of Epidemiology and Community Health, University of Minnesota School of Public Health, 1300 S. 2nd Street, Minneapolis, MN 55454 USA; Department of Preventive Medicine, Northwestern University Feinberg School of Medicine, 680 N. Lake Shore Drive, Chicago, IL 60611 USA; Department of Nutrition and Obesity, The University of Texas School of Public Health, 1200 Pressler St, Houston, TX 77030 USA; Department of Epidemiology, University of California, Berkeley, School of Public Health, 50 University Hall #7360, Berkeley, CA 94720 USA; Division of Psychiatry and Behavioral Sciences, Johns Hopkins School of Medicine, 5300 Alpha Commons Drive, Baltimore, MD 21224 USA; Division of Endocrinology and Metabolism, Johns Hopkins School of Medicine, 1830 E. Monument St, Suite 333, Baltimore, MD 21287 USA

## Abstract

**Background:**

The cross-sectional area of total muscle mass has been reported to decrease by about 40% for those 20–60 years of age. Depressive symptoms may discourage motivation to engage in physical activity such as strength training shown to negate muscle loss. Inflammation related to depressive symptoms may also contribute to muscle atrophy. Physiological differences by sex and race/ethnicity may also modify the association between depression and muscle mass. Evidence on the relationship between depression (or depressive symptoms) and adiposity has been mounting; however, little is known about the depressive symptoms-muscle mass association. We sought to determine the association between elevated depressive symptoms (EDS) and lean muscle mass and whether this varies by sex and race/ethnicity.

**Methods:**

Evaluating 1605 adults (45–84 years of age) from the Multi-ethnic Study of Atherosclerosis Abdominal Body Composition, Inflammation and Cardiovascular Disease Study, we examined the cross-sectional association between EDS (Center for Epidemiologic Studies for Depression Scale score ≥ 16 and/or antidepressant use) and computed tomography-measured abdominal lean muscle mass using linear regression. Muscles were evaluated as a whole and by functionality (locomotion vs. stabilization/posture). Covariates included height, body mass index, sociodemographics, comorbidities, inflammatory markers and health behaviors (pack-years of smoking, alcohol locomotion compared to men, total intentional exercise, daily caloric intake). Sex and race/ethnicity were assessed as potential modifiers. Statistical significance was at a p < 0.05 for main effects and < 0.20 for interaction.

**Results:**

Men with elevated depressive symptoms had 5.9 cm^2^ lower lean muscle mass for locomotion compared to men without EDS, fully-adjusted (CI = −10.5, −1.4, *p* = 0.011). This was statistically significantly different from the null finding among women (interaction *p* = 0.05). Chinese participants with EDS had 10.2 cm^2^ lower abdominal lean muscle mass for locomotion compared to those without EDS (fully-adjusted, CI = −18.3, −2.1, *p* = 0.014), which was significantly different from the null relationship among White participants (interaction *p* = 0.04). No association was observed between elevated depressive symptoms and muscle for stabilization/posture evaluating the whole population or stratified by sex or race/ethnicity.

**Conclusions:**

In the presence of elevated depressive symptoms, men and Chinese participants may have lower muscle mass, particularly for locomotion.

**Electronic supplementary material:**

The online version of this article (doi:10.1186/s12888-015-0604-9) contains supplementary material, which is available to authorized users.

## Background

In the year 2000, sarcopenia, a degenerative loss of muscle mass and strength commonly observed in older populations, accounted for $18.5 billion in healthcare costs [1]. Because of its association with diabetes [2], mortality [3] and frailty [4] among the elderly, identification of high risk groups is necessary to help prevent the development or worsening of these conditions.

Sarcopenia has been found to increase with age [5, 6]. However, even among older adults (>60 years of age), the prevalence varies from 8-40 % which suggests a role for health behaviors or clinical factors [7]. For example, progression of sarcopenia can be attenuated by physical activity, but depressive symptoms may decrease motivation to exercise [8, 9]. In addition, depressive symptoms may be related to inflammation [10], which may contribute to further muscle loss [11, 12]. Only one study has shown the relationship between depression and muscle mass independently of fat mass, and this was limited to a population of Korean elders [13]. Although a statistically significant association between depression and lower lean muscle mass was found among men but not women, differences by race/ethnicity in this study could not be assessed.

There are reasons to suspect sex as a modifier of this association. Uncontrolled, elevated inflammatory markers (e.g. interleukin-6 [IL-6], tumor necrosis factor-alpha) can occur via a dysfunctional hypothalamic pituitary adrenal axis (e.g. during chronic stress, depression) [14]. In response to stress, a study found women to have greater sensitivity to adrenal cortex stimulation which may lead to greater inflammation compared to men [15]. In contrast, other studies have reported higher cortisol responses in men compared with women after exposure to acute real-life psychological stress [16, 17], which might contribute to excessive inflammation, and in turn, promote greater muscle deterioration. It has been proposed that depression in men may manifest as increased alcohol consumption. This increased intake may result in greater risk for alcohol-induced myopathy and lead to less physical activity, further lowering lean muscle mass [18].

Race/ethnic differences in the relationship between depressive symptoms and muscle mass may also exist. Depression can induce an inflammatory response that may contribute to muscle atrophy [10, 19]. Depression-induced stress can cause inflammation, a response that has been shown greater among Black than White women [20]. Early life adversity has also been associated with high inflammation levels in midlife for Blacks, but not for Whites [21]. Further, the loss of interest in pleasurable activities, including exercise, are common among depressed individuals [22]. Compared with Whites, Blacks and Hispanics are, on average, less active during leisure time [22]. Thus, in the presence of depression, these ethnic minorities may endure faster muscle atrophy in the setting of low baseline physical activity [23]. On the other hand, and as noted above, excessive alcohol consumption can lead to myopathy [18], and greater alcohol consumption among Whites compared to Blacks and Asians may put the minority groups at lower risk for alcohol-induced myopathy [24]. Conversely, less tolerance from alcohol due to alcohol flush reactions common among Asians and smaller body stature may trigger myopathy at a lower dose of consumption compared to other ethnic groups [25, 26].

Evidence on depression and subsequent increase in adiposity has been well established [27–31]; however, little is known about the potential depressive symptoms-muscle mass association, especially among different racial/ethnic groups. In the current study, we evaluated this relationship and assessed potential modification by sex and race/ethnicity using a diverse, multi-racial/ethnic population.

## Methods

### Population and study design

The Multi-ethnic Study of Atherosclerosis (MESA) is a multi-center, community-based cohort study to examine the prevalence, correlates and progression of subclinical cardiovascular disease [32]. There were 6814 men and women aged 45–84 years who identified themselves as White, Chinese, Black or Hispanic, and were free of overt clinical cardiovascular disease recruited from 6 US communities: Baltimore City and Baltimore County, Maryland; Chicago, Illinois; Forsyth County, North Carolina; Los Angeles County, California; Northern Manhattan and the Bronx, New York; and St. Paul, Minnesota. The first exam took place between 2000 and 2002, the second between 2002 and 2004, the third between 2004 and 2005, the fourth between 2005 and 2007, and the fifth visit between 2010 and 2012. The current cross-sectional analyses utilized outcome data obtained from either the second or third exam. Additional details about the design and objectives of MESA have been described elsewhere [32]. The study was approved by the institutional review boards of each institution (i.e. Columbia University, New York; Johns Hopkins University, Baltimore; Northwestern University, Chicago; the University of California, Los Angeles, Los Angeles; University of Minnesota, Twin Cities; and Wake Forest University, Winston Salem), and written informed consent was obtained from each participant.

### Computed Tomography (CT) Scan

Computed tomography (CT) scans were obtained during visits 2 or 3 on a subset of 1968 subjects who enrolled in an ancillary study to determine the presence and extent of calcified atherosclerosis in the abdominal aorta. Details of the CT scans have been previously published [33]. Abdominal body composition was determined in 1944 participants enrolled in the MESA Body Composition, Inflammation and Cardiovascular Ancillary Study using these scans. In brief, six selected slices from the L2-L5 vertebral spaces (i.e. two each at L2–L3, L3–L4, and L4–L5) were obtained for measurement of abdominal lean muscle mass (in cm^2^). Using the Medical Imaging Processing Analysis and Visualization software (MIPAV, version 4.1.2), two trained analysts evaluated each CT scan independently at a centralized reading center (UCSD, La Jolla, CA). The Hounsfield Unit range used to classify tissue was: 0 ≤ muscle pixel ≤ 100. A summary measurement of all six scans was used to define lean muscle mass in the abdomen.

In addition to being assessed as a whole, muscle mass was classified as locomotion (psoas) or stabilization/posture (rectus abdominus, obliques, paraspinal) to explore whether elevated depressive symptoms (EDS) was differentially related to muscle functional group. Due to limitations of the scanner field of view (FOV), some measurements for rectus abdominus and obliques may be underestimated. The relatively central positioning of the paraspinous allowed for complete measurement within the available FOV. Measurements for paraspinous highly correlated with that for rectus abdominus and obliques, and, thus, were used as a proxy for muscles for stabilization/posture.

### Assessment of depressive symptoms

Depressive symptoms were assessed during visit 3 using the Center for Epidemiologic Studies Depression (CES-D) Scale, a 20-item questionnaire developed to assess past week’s depressive symptoms among community populations [34], administered in English, Spanish, Cantonese and Mandarin. Participants were asked to rate each item on a scale from 0–3 (range of total scores = 0-60). Although the CES-D is not an assessment of clinical depression, a score of ≥16 has been found consistent with at least mild-to-moderate depression or dysthymia [35]. Using this cut-off, sensitivity and specificity for major depression in the past year had been reported as 70.6 % and 88.0 %, respectively [35]. The internal consistency of CES-D has ranged between a Cronbach’s alpha of 0.84 and 0.93 [36], and has been found comparable in European-American, African-American, Mexican-American and Chinese-American groups [34, 37, 38]. The CES-D has been used widely in cross-cultural epidemiological studies conducted with validated Spanish [39] and Chinese versions [38].

The use of antidepressants may likely be indicative of the presence of more severe depressive symptoms, which may not be captured by measuring CES-D alone. For this reason, EDS was defined as having a CES-D score ≥ 16 and/or the use of antidepressants. Depressive symptoms were also modeled as a continuous variable.

### Covariates

Using standard protocols as previously described [32], individual-level covariates were collected including height, body mass index (BMI), sociodemographics (age, sex, race/ethnicity, marital status, education [high school graduate or below, high school graduate, beyond high school], annual income, study site), inflammatory markers (interleukin-6 [IL-6], C-reactive protein [CRP]), health behaviors (pack-years of smoking, alcohol consumption per week, total intentional exercise) and co-morbidities (diabetes, cancer, hypertension). Weight and height were measured using a balance beam scale and a stadiometer, respectively, with participants wearing light clothing. BMI was calculated as weight (kg) per height squared (m^2^). IL-6 and CRP were measured using ultra-sensitive ELISA (Quantikine HS Human IL-6 Immunoassay; R&D Systems, Minneapolis, MN) and the BNII nephelometer (N High Sensitivity CRP; Dade Behring Inc., Deerfield, IL), respectively [40]. Total intentional exercise was determined using a 28-item Typical Week Physical Activity Survey [32, 41]. Physical activity was summarized as the metabolic equivalent task of minutes per week spent in moderate or vigorous household, outdoor, sporting, conditioning and volunteer activities. Type 2 diabetes was defined by fasting plasma glucose ≥ 126 mg/dl and/or use of medications for diabetes [42]. Self-reported cancer included a doctor’s diagnosis for prostate, breast, lung, colon, blood, non-melanoma skin cancer, or other cancer. Hypertension was defined by a systolic blood pressure ≥ 140 mmHg, a diastolic blood pressure ≥ 90 mmHg or use of anti-hypertensive medications [43]. Blood pressure was measured in a seated position three times, and the average of the last two measurements was used. Covariates were chosen based on findings from prior research and clinical evidence.

Variables with measurements available during visits (2 or 3) corresponding to the abdominal CT scan and not missing in abundance were used in the analyses: age, height, BMI, total intentional exercise and antidepressant use. Baseline values were used for other covariates.

### Statistical analyses

There were 1944 individuals from whom abdominal body composition was determined. For the current analysis, participants were excluded for the following reasons: incomplete muscle data (*n* = 5), missing CES-D scores at visit 3 (*n* = 82) and missing covariate values (*n* = 252). As such, a total of 1605 participants were included.

We compared the distribution of baseline characteristics by depressive symptom status using student’s ‘t’ tests for continuous variables and chi-square tests for categorical variables. Continuous variables were presented as a mean and standard deviation when normally distributed and as a median and interquartile range when non-normally distributed. Categorical variables were expressed as frequency and percentage (n [%]). Normality of continuous variables were evaluated with the use of kernel density plots and plots comparing quantiles of each of these variables versus that of a normal distribution. For non-normally distributed continuous variables, the Wilcoxon Rank-Sum Test was used for the two-group comparison.

Linear regression models were used with lean muscle mass (overall, locomotion, and stabilization/posture) as the dependent variable and depressive symptoms status as the main independent variable adjusting for a series of covariates. To account for body compartment size, analyses were adjusted for age, height and BMI as done in previous studies [13, 30]. After this baseline adjustment (Model 1), Model 2 additionally included adjustment for sociodemographics. Adjustments for inflammatory markers, health behaviors and comorbidities were also performed in addition to baseline adjustment to assess potential mediation (Model 3) [10–12, 18, 19, 22, 23]. The fully-adjusted model included all covariates (Model 4).

To evaluate modification by sex, a 1^st^ order interaction term was created by multiplying depressive symptoms status with sex. To assess modification by race/ethnicity, three dummy variables were generated, with the White population as the referent group, each of which was multiplied by depressive symptoms status to create three 1^st^ order interaction terms and both the individual as well as the 3-degrees of freedom interaction test were assessed. Significance was determined using Wald tests obtained from analyzing fully-adjusted models.

Analyses were completed using STATA (StataCorp. 2012. *Stata Statistical Software: Release 12.* College Station, TX). Statistical significance was considered at a two-sided type 1 error <0.05 for main effects. A p < 0.20 was used to determine the presence of interaction as in prior studies [44–46].

## Results

Table 1 illustrates the population characteristics by EDS status. Overall, 18.4 % of the sample had EDS, with a median CES-D score of 18.0 (interquartile range = 9, 23). Among those with EDS, 45.2 % utilized antidepressants. They were also, on average, younger, more likely to be women, of Hispanic descent and single, and were less educated. EDS were also significantly associated with shorter stature, greater prevalence of overweight/obesity based on both waist circumference and BMI, and lower lean muscle mass (overall, locomotion and stabilization/posture).

**Table 1:** Baseline population characteristics by Elevated Depressive Symptoms (EDS, CES-D ≥16 and/or antidepressant use) status (*N* = 1605)

	No EDS	EDS	*p*-value
	(*n* = 1306, 81.4 %)	(*n* = 299, 18.6 %)	
CES-D (median [IQR])	4 (1, 8)	18 (9, 23)	<0.001*
Antidepressant Use (% Yes)	NA	135 (45.2)	NA
Demographics			
Age, in years (mean [SD])	64.7 (9.6)	62.9 (9.0)	0.004*
Sex (% Women)	626 (47.9)	197 (65.9)	<0.001*
Race/ethnicity (%)			0.001*
White	535 (41.0)	132 (44.2)	
Chinese	200 (15.3)	32 (10.7)	
Blacks	273 (20.9)	42 (14.1)	
Hispanics	298 (22.8)	93 (31.1)	
Marital Status (%)			<0.001*
Married	896 (68.6)	167 (55.9)	
Widowed/Divorced/Separated	331 (25.3)	98 (32.8)	
Single	79 (6.1)	34 (11.4)	
Education (%)			0.077
Less than High School	216 (16.5)	64 (21.4)	
High School	212 (16.2)	53 (17.7)	
College or greater	878 (67.2)	182 (60.9)	
Income (%)			0.137
<$25,000	365 (33.7)	101 (38.6)	
≥ $25,000 and < $50,000	382 (35.3)	92 (35.1)	
≥ $50,000 and < $75,000	218 (20.1)	52 (19.9)	
≥ $75,000	118 (10.9)	17 (6.5)	
Inflammatory Markers			
IL-6, in pg/mL (median [IQR])^a^	1.1 (0.7, 1.7)	1.2 (0.8, 1.9)	0.058
CRP, in mg/L (median [IQR])^a^	1.7 (0.8, 3.9)	2.1 (0.9, 4.6)	0.082
Health Behavior			
Alcohol Consumption/Week (mean [SD])	4.0 (7.7)	3.9 (8.5)	0.999
Pack-years of Smoking (mean [SD])	11.5 (27.4)	10.8 (19.1)	0.705
Total Intentional Exercise, in met-min/wk (median [IQR])^b^	900 (210, 2118)	1477 (1100, 2055)	0.180
Comorbidities (% Yes)			
Diabetes	132 (10.1)	35 (11.7)	0.414
Cancer	108 (8.3)	20 (6.7)	0.363
Hypertension	548 (52.0)	138 (46.2)	0.186
Anthropometry			
Height (mean, [SD])	166.7 (9.8)	164.0 (9.7)	<0.001*
Waist Circumference, in cm (mean [SD])	96.7 (13.4)	99.6 (15.0)	0.001*
Overweight/Obese (%)^b^	634 (48.6)	187 (62.5)	<0.001*
BMI, in kg/m^2^ (mean [SD])	27.5 (4.9)	28.9 (5.5)	<0.001*
Overweight/Obese (%)^b^	886 (67.8)	222 (74.3)	0.031*
Weight, in lb (mean, [SD])	169.3 (35.4)	171.6 (37.5)	0.313
Lean Muscle Mass, in cm^2^ (mean, SD])			
Combined	390.1 (104.6)	362.7 (93.6)	<0.001*
Locomotion	115.3 (37.5)	105.1 (33.1)	<0.001*
Stabilization	274.8 (74.0)	257.6 (68.6)	<0.001*

* Significant p-value <0.05

^a^IQR = Interquartile Range

^b^Overweight/Obese: Waist Circumference, > 88 cm (women) and > 102 cm (men); BMI ≥ 25 kg/m^2^

**Fig. 1:**
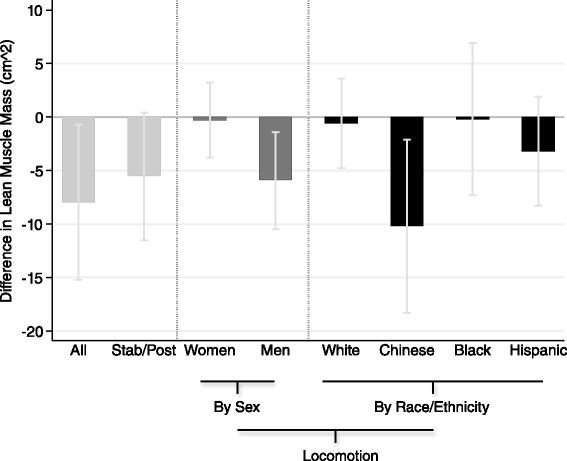
Graph of Difference in Lean Muscle Mass Between Elevated Depressive Symptoms Status. Stab/Post = Lean muscle mass for stabilization/posture. * Significant interaction at *p* < 0.20 (referent group for interaction by race/ethnicity: White). NOTE: Overall 3-degrees of freedom interaction p-value for race/ethnicity (reference: White) = 0.19: Chinese vs. White = 0.04; Black vs. White = 0.93; Hispanic vs. White = 0.43; interaction p-value for sex = 0.05. Estimates are from models adjusted for age, height, BMI, sex, race/ethnicity, marital status, education, income, study site, comorbidities, inflammatory markers, alcohol consumption per week, pack-years of smoking and total intentional exercise

Table 2 summarizes the results of the main analyses. Evaluating the overall sample population, EDS was inversely associated with lean muscle mass (overall, locomotion or stabilization/posture) after adjustment for age, height and BMI. Additional adjustment for sociodemographics attenuated the findings to about half the estimate. Adjustment for inflammatory markers and other health behaviors, in addition to age, height and BMI, negligibly altered the estimate compared to the baseline model. Fully-adjusted, participants with EDS had 8.0 cm^2^ significantly lower lean muscle mass compared to those without EDS. The relationship between EDS and lean muscle mass for locomotion varied by sex (fully-adjusted interaction *p* = 0.05) and race/ethnicity (fully-adjusted interaction *p* = 0.19), which was driven by the difference comparing the results between White and Chinese participants (fully-adjusted interaction *p* = 0.04). Figure 1 illustrates the association between EDS and lean muscle mass (overall and stabilization/posture), as well as between EDS and lean muscle mass for locomotion by sex and race/ethnicity. EDS was not statistically related to lean muscle mass for stabilization/posture, nor was there interaction by either sex or race/ethnicity in this association.

**Table 2:** Lean muscle mass (locomotion, stabilization/posture) difference (95 % confidence interval [CI]) between elevated depressive symptoms status (EDS, CES-D ≥ 16 and/or antidepressant use)^a^

Stratification	Adjustment Models (Difference [95 % CI])^b^			
	Model 1	Model 2	Model 3	Model 4
ALL LEAN MUSCLE MASS (in cm^2^, *N* = 1605)	−17.8 (−26.9, −8.8)*	−7.6 (−14.9, −0.3)*	−18.9 (−27.7, −10.0)*	−8.0 (−15.2, −0.7)*
LOCOMOTION				
All (in cm^2^, *N* = 1,605)	−6.2 (−9.7, −2.7)*	−2.5 (−5.3, 0.4)	−6.5 (−9.9, −3.1)*	−2.4 (−5.2, 0.4)
By Sex (in cm^2^)				
Women (*n* = 823, 51.3 %)	−1.8 (−5.6, 1.9)	−0.3 (−3.9, 3.2)	−1.8 (−5.5, 1.9)	−0.3 (−3.8, 3.2)
Men (*n* = 782, 48.7 %)	−5.4 (−10.2, −0.5)	−6.1 (−10.7, −1.5)*	−5.3 (−10.0, −0.5)*	−5.9 (−10.5, −1.4)*
By Race/ethnicity (in cm^2^)				
White (*n* = 667, 41.6 %)	−5.1 (10.3, 0.1)	−0.4 (−4.6, 3.9)	−5.7 (−10.8, −0.7)*	−0.6 (−4.8, 3.6)
Chinese (*n* = 232, 14.5 %)	−14.5 (−24.6, −4.4)*	−9.8 (−18.0, −1.6)*	−15.4 (−25.3, −5.6)*	−10.2 (−18.3, −2.1)*
Black (*n* = 315, 19.6 %)	−1.6 (−10.5, 7.2)	−0.2 (−7.4, 7.0)	−2.1 (−10.7, 6.5)	−0.2 (−7.3, 6.9)
Hispanic (*n* = 391, 24.4 %)	−5.4 (−11.8, 0.9)	−3.9 (−9.0, 1.3)	−4.7 (−10.9, 1.4)	−3.2 (−8.3, 1.9)
STABILIZATION/POSTURE				
All (in cm^2^, *N* = 1605)	−11.6 (−18.4, −4.8)*	−5.1 (−11.4, 0.8)	−12.4 (−19.1, −5.7)*	−5.5 (−11.5, 0.4)

^a^Significant interaction at a p-value < 0.20: Locomotion Muscles: (1) Ethnic minority vs. White = 0.19: Chinese vs. White = 0.04; Black vs. White = 0.93; Hispanic vs. White = 0.43; (2) by sex = 0.05; Stabilization Muscles: (1) Ethnic minority vs. White = 0.81: Chinese vs. White = 0.87; Black vs. White = 0.33; Hispanic vs. White = 0.76; (2) by sex = 0.22

^b^Model 1 = Adjusted for age, height, BMI (main effects for sex or race/ethnicity were evaluated in this model when assessing interaction for sex or race/ethnicity, respectively; otherwise they were included in model 2); Model 2 = Adjusted for Model 1, sex, race/ethnicity, marital status, education, income, study site, and comorbidities (diabetes, cancer, hypertension); Model 3 = Adjusted for Model 1, inflammatory markers (IL-6, CRP), other health behaviors (alcohol consumption per week, pack-years of smoking, and total intentional exercise); Model 4 = Fully-adjusted

*Significant at a *p*-value < 0.05 for main effects

EDS were not significantly associated with lean muscle mass for locomotion in women. In contrast, among men, there was a significant negative association between EDS and lean muscle mass for locomotion. Those with EDS had 5.4 cm^2^ lower lean muscle mass for locomotion after adjustment for age, height and BMI (Table 2). This estimate slightly increased following additional adjustment for sociodemographics, but negligibly changed following additional adjustment for inflammatory markers, health behaviors, and comorbidities. Fully-adjusted, men with EDS had 5.9 cm^2^ lower lean muscle mass for locomotion compared to men without EDS (Table 2).

Interaction between EDS and race/ethnicity was driven by the difference between White and Chinese participants (*p* = 0.04). Minimally adjusted for age, height and BMI, Chinese participants with EDS had 14.5 cm^2^ lower lean muscle mass for locomotion compared to their counterparts without EDS. Additional adjustment for sociodemographics attenuated the estimate, but not after additional adjustment for inflammatory markers, health behaviors and comorbidities. Fully-adjusted, Chinese participants with EDS had 10.2 cm^2^ significantly lower lean muscle mass for locomotion compared to Chinese participants without EDS. This estimate was statistically different than the estimate found among White participants (fully-adjusted interaction *p*-value = 0.04) (Table 2). The association between EDS and lean muscle mass for locomotion among Blacks and Hispanics were not statistically significant nor different from White participants.

Depressive symptoms modeled continuously were inversely and significantly associated with lean muscle mass (fully adjusted: whole [beta coefficient = −0.53, CI = −0.9, −0.2]; locomotion [beta coefficient = −0.2, CI = −0.3, −0.03]; stabilization/posture [beta coefficient = −0.4, CI = −0.7, −0.04]). There was no interaction by race/ethnicity; however, all associations varied by sex with men having lower muscle mass per unit increase in depressive symptoms (data not shown). In addition, the inverse relationship was only significant among men (all p-interaction <0.1, data not shown).

Limiting to participants with visit 3 CT scans, the pattern of association produced similar results; however, no significant interaction by either sex or race/ethnicity was found (Table S1).  Limiting to participants with visit 2 CT scans produced similar trends as evaluating the study using both visits 2 and 3 CT scans combined (Table S2).

## Discussion

In this study, EDS were significantly associated with overall lean muscle mass after adjustment for height, BMI, sociodemographics, inflammatory markers, health behaviors and comorbidities. The relationship between EDS and lean muscle mass for locomotion significantly varied by sex and race/ethnicity. Men with EDS had significantly lower lean muscle mass for locomotion than men without EDS; whereas, among women, this association was not significant. EDS among Chinese participants were also associated with lower lean muscle mass for locomotion, which was statistically different from the null association among Whites. There was neither a significant association between EDS and lean muscle mass for stabilization/posture nor modification of this relationship by sex or race/ethnicity.

Only one study of an elderly Korean population has investigated whether lean muscle mass is related to EDS [13]. As such, the current study is the first known to assess this relationship using a multi-racial/ethnic US population. The results of modification by sex support the findings by Kim et al. [13], showing a significant negative association between depressive symptoms and lean muscle mass among men, and the null relationship among women.

The resulting modification by sex may be explained by differences in body image, level of inflammation and alcohol consumption. In Westernized societies, a bulkier, muscular physical appearance may be more ideal for men compared to women who may prefer a smaller build [47–49]. Hence, for men, musculature may be more related to depression with regards to body image dissatisfaction, which is supported by our findings. Greater inflammatory response may also explain muscle loss in men but not in women in the presence of EDS. Studies have reported higher cortisol responses in men compared to women after exposure to acute real-life psychological stress [16, 17], which might lead to greater inflammation, and in turn, lower muscle mass. Men have also been proposed to manifest depression through increased alcohol consumption which, in excess, may cause alcohol-induced myopathy, discouraging physical activity and further deteriorating lean muscle mass [18]. Adjustment for inflammatory markers and amount of alcohol consumption did not negate the significant association; however, inadequate adjustment may occur as these variables were baseline values. Although inflammatory markers were measured at visits 2 and 3, baseline values were chosen to maintain sample size, particularly in the assessment of modification in the current study.

It is possible that the difference between sexes may be affected by the amount of available muscle mass. As the women in our sample may be largely postmenopausal, sex steroids which maintain muscle mass may already be at low levels; whereas for men, testosterone decline is gradual, allowing for greater muscle mass at a longer duration [50]. If EDS were associated with lower muscle mass, greater detection would be found among men in this cohort who may have had greater muscle reserve compared to women who may have had probable low lean muscle mass at study initiation.

Asians in general have the lowest level of fat-free muscle mass compared to Whites, Blacks and Hispanics [51, 52]. Higher lean muscle mass is associated with improved insulin sensitivity [53], which, in contrast, is negated by increased adiposity [54]. Thus, the accumulation of body fat unaccompanied by a simultaneous increase in lean muscle mass among Asians may subject them to a greater risk for insulin resistance and type 2 diabetes compared to other race/ethnic groups. As type 2 diabetes is associated with increased risk for EDS [55], low levels of lean muscle mass followed by increased adiposity and type 2 diabetes may thus lead to increased depressive symptomatology. This may additionally explain the significant negative association between lean muscle mass for locomotion and depressive symptomatology among the Chinese cohort in the current study. Conversely, depression-induced type 2 diabetes may also lead to muscle atrophy [56]. In general, ethnic minorities have a higher propensity for type 2 diabetes compared to Whites [57], and the additional risk of type 2 diabetes attributable to EDS may explain the lower lean muscle between those with and without EDS comparing the Chinese and Hispanic cohorts to White participants. The potential increased risk for insulin resistance and type 2 diabetes among Asians previously discussed may explain the greater disparity between Chinese and White participants in this study. Adjustment for the presence of diabetes, however, only minimally changed the estimate in all models.

The negligible attenuation following adjustment for potential mediators in this study may likely be a result of using baseline covariates. Longitudinal studies are needed in which the timing of mediation assessment is between that of exposure and outcome. Studies of other factors (e.g. body image, sex hormones) that may help explain the mechanism through which we find differences in sex and race/ethnicity in the association between depressive symptoms and lean muscle mass, particularly for locomotion, are also warranted.

Our study shows a differential relationship between depressive symptoms and lean muscle mass by functionality with statistical significance found only when evaluating lean muscle mass for locomotion, but not for stabilization/posture. EDS may hinder physical activities that promote movement (e.g. walking, running, aerobics), which may lead to lower lean muscle mass for locomotion. In contrast, muscles of stabilization/posture are commonly used in daily activities (e.g. standing, sitting) and, as such, may likely be maintained despite the presence of EDS. Studies that further evaluate these hypotheses are warranted.

The current study possessed certain limitations. First, the cross-sectional nature of the study did not allow for the assessment of temporality. Reverse causality is possible, as well as a bi-directional relationship. Further, cross-sectional studies are subject to survival bias. Future longitudinal studies are necessary to address the direction of association between depressive symptoms and lean muscle mass, and possibly assess and account for differences in survival. Second, depressive symptoms were not assessed concurrently in individuals who had CT scans at visit 2. In subsidiary analyses, however, where individuals with CT scans at visit 2 and visit 3 were analyzed separately, the associations of depressive symptoms with lean muscle mass were similar in magnitude and direction (see Tables S1 and S2). Third, it might be more important to assess resistance training (which was not measured in the current study) rather than participation in moderate or vigorous exercise as this is more specific for building muscle mass; thus, in future studies adjustment specifically for resistant training should be included. Fourth, a few potential confounders/mediators were assessed using baseline values due to limited availability during visits 2 and 3, resulting in residual confounding and limitation in assessing mediation. Further, psoas is used as a surrogate for unmeasured larger muscle mass such as quadriceps, hamstrings and calves which may be more directly associated with depressive symptoms with regards to muscle atrophy due to lower physical activity. Not using these muscle types, however, only renders our result conservative. Finally, the use of CES-D to assess depressive symptoms is not necessarily indicative of clinical depression. The CES-D, however, has been shown in literature to have good reliability and concurrent, criterion and construct validity [34, 58, 59]. In addition, the reliability of the CES-D is comparable in European American, African American, Mexican-American and Chinese American groups [37, 60, 61]. The use of antidepressant medication was also a criteria for the presence of EDS; however, differences in access and utilization by race/ethnicity may identify more individuals in one group over another. Further, we assumed that antidepressant use was specifically prescribed to treat severe depressive symptoms. Misclassification may be minimal, however, as majority of antidepressant use is to treat EDS.

The current study also possessed inherent strengths. MESA provided a number of covariates to account for potential confounding/mediation. Further, MESA is unique in including four race/ethnic groups which is more inclusive of the racial profile in the US. Unlike prior studies that have evaluated muscle mass, we were able to evaluate a greater area using >2 images which may provide a more robust estimate of the relationship between depressive symptoms and muscle mass.

Future longitudinal studies are necessary to (a) elucidate temporality of this association; (b) understand the biological and behavioral mechanisms of the association that should be targeted for potential interventions, with particular attention to sex and racial/ethnic differences; and (c) determine the clinical relevance of the observed difference in lean muscle mass for the purpose of monitoring intervention effectiveness to improve muscle mass in the setting of depression.

## Conclusions

Our results provide evidence that depressive symptoms are significantly related to lean muscle mass and that these associations vary by sex and race/ethnicity. Although longitudinal assessments are necessary to delineate association direction, our findings provide support for sex- and race/ethnic-specific interventions (i.e. to decrease depressive symptoms or increase muscle mass) targeting men and Chinese-Americans.

## Additional files

Additional file 1: Table S1. Lean muscle mass (locomotion, stabilization/posture) difference between elevated depressive symptoms status (EDS, CES-D>16 and/or antidepressant use)^a^ – Visit 3 CT-scans. (DOC 110 kb) Additional file 2: Table S2. Lean muscle mass (locomotion, stabilization/posture) difference between elevated depressive symptoms status (EDS, CES-D>16 and/or antidepressant use)^a^ – Visit 2 CT-scans. (DOC 137 kb)

## Abbreviations

BMI: Body mass index
CES-D: Center for epidemiologic studies – depression
CRP: C-reactive protein
CT: Computed tomography
EDS: Elevated depressive symptoms
FOV: Field of view
IL-6: Interleukin-6
MESA: Multi-ethnic study of atherosclerosis
MIPAV: Medical imaging processing analysis and visualization software
