# Supplementary material for: Sex and race/ethnic disparities in the cross-sectional association between depressive symptoms and muscle mass: the Multi-ethnic Study of Atherosclerosis
Source: BMC Psychiatry. 2015 Sep 18;15:221. doi: 10.1186/s12888-015-0604-9 (PMC4574470; doi:10.1186/s12888-015-0604-9)
Supplement: Additional file 1: Table S1. — Lean muscle mass (locomotion, stabilization/posture) difference between elevated depressive symptoms status (EDS, CES-D>16 and/or antidepressant use)a – Visit 3 CT-scans. (DOC 110 kb) [file 12888_2015_604_MOESM1_ESM.doc]

**Additional file 1: Table S1. Lean Muscle (Locomotion, Stabilization) Difference Between Elevated Depressive Symptoms Status (EDS, CES-D>16 and/or Antidepressant Use)^a^ – Visit 3 CT-scans**

| **Stratification** | **Adjustment Models ^b^** | | | |
| --- | --- | --- | --- | --- |
|  | **Model 1** | **Model 2** | **Model 3** | **Model 4** |
| ALL LEAN MUSCLE  (in cm^2^, N=1 038) | -17.9 (-28.9, -6.9)* | -7.5 (-16.7, 1.7) | -18.0 (-28.8, -7.2)* | -7.8 (-16.9, 1.3) |
|  |  |  |  |  |
| LOCOMOTION |  |  |  |  |
| All (in cm^2^, N=1 038) | -7.5 (-11.8, -3.3)* | -3.8 (-7.3, -0.2)* | -7.3 (-11.5, -3.2)* | -3.7 (-7.2, -0.2)* |
|  |  |  |  |  |
| STABILIZATION |  |  |  |  |
| All (in cm^2^, N=1 038) | -10.4 (-18.8, -2.1)* | -3.7 (-11.2, 3.8) | -10.7 (-18.9, -2.4)* | -4.1 (-11.6, 3.4) |

^a^ Significant interaction at a p-value < 0.20: Locomotion Muscles: (1) Ethnic minority vs. White=0.48: Chinese vs. White=0.20; Black vs. White=0.74; Hispanic vs. White=0.41; (2) by sex = 0.61; Stabilization Muscles: (1) Ethnic minority vs. White=0.63: Chinese vs. White=0.81; Black vs. White=0.22; Hispanic vs. White=0.95; (2) by sex = 0.32

^b^ Model 1 = Adjusted for age, height, BMI (main effects for sex or race/ethnicity were evaluated in this model when assessing interaction for sex or race/ethnicity, respectively; otherwise they were included in model 2); Model 2 = Adjusted for Model 1, sex, race/ethnicity, marital status, education, income, study site; Model 3 = Adjusted for Model 1, inflammatory markers (IL-6, CRP), other health behaviors (alcohol consumption per week, pack-years of smoking, total intentional exercise), comorbidities (diabetes, cancer, hypertension); Model 4 = Fully-adjusted

* Significant at a p-value < 0.05 for main effects
