# Supplementary material for: Sex and race/ethnic disparities in the cross-sectional association between depressive symptoms and muscle mass: the Multi-ethnic Study of Atherosclerosis
Source: BMC Psychiatry. 2015 Sep 18;15:221. doi: 10.1186/s12888-015-0604-9 (PMC4574470; doi:10.1186/s12888-015-0604-9)
Supplement: Additional file 2: Table S2. — Lean muscle mass (locomotion, stabilization/posture) difference between elevated depressive symptoms status (EDS, CES-D>16 and/or antidepressant use)a – Visit 2 CT-scans. (DOC 137 kb) [file 12888_2015_604_MOESM2_ESM.doc]

**Additional file 2: Table S2. Lean Muscle (Locomotion, Stabilization) Difference Between Elevated Depressive Symptoms Status (EDS, CES-D>16 and/or Antidepressant Use)^a^ – Visit 2 CT-scans**

| **Stratification** | **Adjustment Models ^b^** | | | |
| --- | --- | --- | --- | --- |
|  | **Model 1** | **Model 2** | **Model 3** | **Model 4** |
| ALL LEAN MUSCLE MASS  (in cm^2^, N=567) | -17.2 (-33.0, -1.4)* | -11.3 (-23.5, 0.9) | -18.1 (-33.8, -2.5)* | -10.5 (-22.6, 1.6) |
|  |  |  |  |  |
| LOCOMOTION |  |  |  |  |
| All (in cm^2^, N=567) | -3.5 (-9.6, 2.6) | -1.0 (-58, 3.7) | -4.2 (-10.2, 1.7) | -1.0 (-5.8, 3.7) |
|  |  |  |  |  |
| By Sex (in cm^2^) |  |  |  |  |
| Women (n=298, 52.6%) | 2.2 (-4.2, 8.6) | 3.5 (-2.5, 9.6) | 1.6 (-4.8, 7.9) | 3.0 (-2.9, 9.0) |
| Men (n=269, 47.4%) | -9.0 (-17.0, -0.9)* | -8.4 (-16.0, -0.7)* | -8.0 (-16.0, -0.02)* | -7.6 (-15.2, 0.01) |
|  |  |  |  |  |
| By Race/ethnicity (in cm^2^) |  |  |  |  |
| White (n=667, 41.6%) | -3.1 (-14.4, 8.2) | 4.1 (-4.9, 13.0) | -7.2 (-18.5, 4.1) | 3.3 (-5.7, 12.4) |
| Chinese (n=232, 14.5%) | -15.5 (-29.8, -1.2)* | -10.3 (-21.7, 1.0) | -15.8 (-29.8, -1.9)* | -10.5 (-21.7, 0.7) |
| Black (n=315, 19.6%) | 0.2 (-12.0, 12.4) | -0.3 (-10.0, 9.4) | -0.8 (-12.7, 11.1) | -1.3 (-10.9, 8.3) |
| Hispanic (n=391, 24.4%) | 1.0 (-9.6, 11.6) | -0.9 (-9.2, 7.4) | 2.9 (-7.4, 13.3) | 0.6 (-7.6, 8.8) |
|  |  |  |  |  |
| STABILIZATION/POSTURE |  |  |  |  |
| All (in cm^2^, N=567) | -13.7 (-25.3, -2.1)* | -10.3 (-20.0, -0.5)* | -13.9 (-25.4, -2.4)* | -9.4 (-19.1, 0.2) |

^a^ Significant interaction at a p-value < 0.20: Locomotion Muscles: (1) Ethnic minority vs. White=0.28: Chinese vs. White=0.06; Black vs. White=0.49; Hispanic vs. White=0.66; (2) by sex = 0.03; Stabilization Muscles: (1) Ethnic minority vs. White=0.93: Chinese vs. White=0.57; Black vs. White=1.00; Hispanic vs. White=0.73; (2) by sex = 0.46

^b^ Model 1 = Adjusted for age, height, BMI (main effects for sex or race/ethnicity were evaluated in this model when assessing interaction for sex or race/ethnicity, respectively; otherwise they were included in model 2); Model 2 = Adjusted for Model 1, sex, race/ethnicity, marital status, education, income, study site; Model 3 = Adjusted for Model 1, inflammatory markers (IL-6, CRP), other health behaviors (alcohol consumption per week, pack-years of smoking, total intentional exercise), comorbidities (diabetes, cancer, hypertension); Model 4 = Fully-adjusted

* Significant at a p-value < 0.05 for main effects
